# Animal Cell Lines as Expression Platforms in Viral Vaccine Production: A Post Covid-19 Perspective

**DOI:** 10.1021/acsomega.3c10484

**Published:** 2024-04-02

**Authors:** S. Furkan Demirden, Ilgin Kimiz-Gebologlu, Suphi S. Oncel

**Affiliations:** Ege University, Bioengineering Department, Izmir, 35100, Turkiye

## Abstract

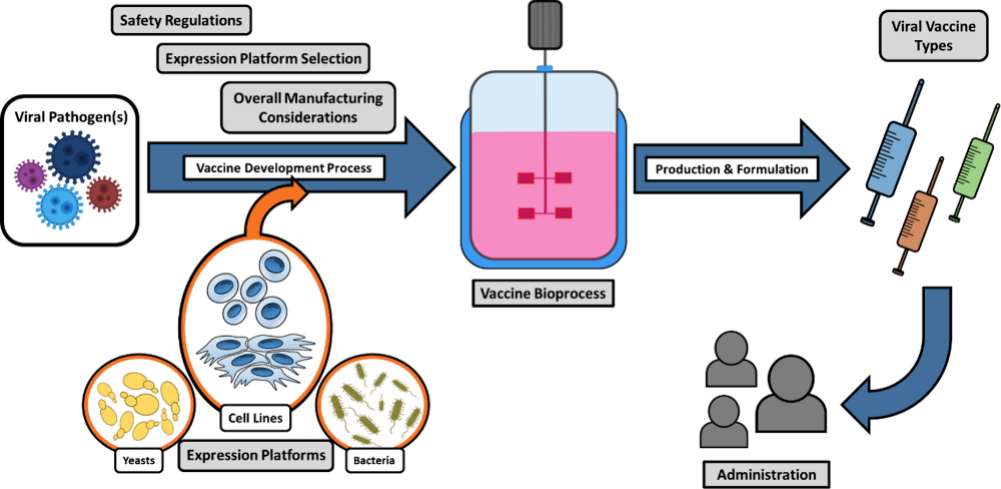

Vaccines are considered the most effective tools for preventing diseases. In this sense, with the Covid-19 pandemic, the effects of which continue all over the world, humanity has once again remembered the importance of the vaccine. Also, with the various epidemic outbreaks that occurred previously, the development processes of effective vaccines against these viral pathogens have accelerated. By these efforts, many different new vaccine platforms have been approved for commercial use and have been introduced to the commercial landscape. In addition, innovations have been made in the production processes carried out with conventionally produced vaccine types to create a rapid response to prevent potential epidemics or pandemics. In this situation, various cell lines are being positioned at the center of the production processes of these new generation viral vaccines as expression platforms. Therefore, since the main goal is to produce a fast, safe, and effective vaccine to prevent the disease, in addition to existing expression systems, different cell lines that have not been used in vaccine production until now have been included in commercial production for the first time. In this review, first current viral vaccine types in clinical use today are described. Then, the reason for using cell lines, which are the expression platforms used in the production of these viral vaccines, and the general production processes of cell culture-based viral vaccines are mentioned. Also, selection parameters for animal cell lines as expression platforms in vaccine production are explained by considering bioprocess efficiency and current regulations. Finally, all different cell lines used in cell culture-based viral vaccine production and their properties are summarized, with an emphasis on the current and future status of cell cultures in industrial viral vaccine production.

## 1 Introduction

Vaccines are one of the most important achievements of humanity against diseases. The concept of “vaccines”, which developed especially with smallpox, has become even more interesting as a result of the diseases that emerged in the historical process which causes pandemics and epidemics. Throughout history, various vaccines have been developed against many diseases based on different scientific methods.^1−3^ As a result of the clinical application of these developed vaccines, this interest in vaccines has become not only a scientific endeavor but also a necessity, with the formation of the concept of public health. Various studies have shown for many years that vaccines are of vital importance in ensuring the healthy continuity of the social, economic, and cultural lives of all individuals in society.^1,3−6^

The concept of vaccines in brief; it can be defined as a biological product that stimulates the immune system of the living thing to which it is applied in order to prevent diseases and triggers a strengthened response when encountering the pathogen.^1,3^ Today, various vaccines have been developed against many pathogens that have been identified as disease agents. The production methods of these vaccines, developed against both bacterial and viral pathogens, vary greatly. Especially in vaccines developed against bacterial pathogens, the disease agent itself or the bacterial toxin associated with the disease can be produced directly, whereas for viral pathogens the process is more complex.^7−10^ This is because viral pathogens, due to their intracellular parasitic nature, do not have the mechanisms to reproduce, so they need a suitable host which they can replicate themselves.^1,6,11,12^ Although in the historical process, live animals such as cattle were first used to propagate the viral agent, the drawbacks of this practice were revealed over time.^2,10,13^ Thus, a driving force has emerged for the use of the animal cell culture technique, in which studies can be carried out at the cellular level by moving away from the structure of a whole organism, in the production of biological products such as vaccines.^4,6,14,15^

In particular, “cell lines” developed based on the integrated progress of genetics, molecular biology, and cell culture techniques stand out in this sense.^6,12,14,15^ In addition to the cell lines that have been used in production with proven safety for many years, improvements are constantly being made in industrial production thanks to their derivatives obtained as a result of various molecular modifications and newly developed cell lines. The reason for this is to meet the ever-increasing demand for biological products and the need for the development of products with higher biological efficiency.^4,12,16,17^ In the production of vaccines, different cell lines are used depending on the type of vaccine desired to be produced, thanks to their “host cell-specific” properties. However, with the new vaccine technologies, traditional cell lines are not sufficient to provide a targeted immunogenic effect. Therefore, the new cell lines are beginning to be used or designed for those which not previously involved in production.^5,6,8,18^ In this respect, in order to protect the continuity of public health, vaccines are constantly produced against the relevant viral pathogens currently in circulation. In addition, in a scenario such as a pandemic, it is necessary to meet the dramatic need for vaccines quickly in order to control the disease and new vaccine technologies with novel cell lines as expression platforms are come forward to meet this demand.^9,17,19^

With the Covid-19 pandemic that affected the whole world and the epidemics that occurred before, interest and need for vaccination has gradually increased. The success of vaccines, which are the most effective defense to prevent epidemic diseases, has been demonstrated by clinical studies on the morbidity and mortality of vaccinated and unvaccinated individuals, as in Covid-19 and many other studies.^20^ In this sense, the Covid-19 pandemic has once again reminded all of humanity of the importance of vaccination and vaccination.^7,21^

In this review, only viral vaccine types are briefly explained and mainly focused on “animal cell lines” as expression platforms in their production. First, traditional viral vaccines that have already been produced commercially for many years and novel viral vaccines which approved and put into commercial use for the first time during the Covid-19 pandemic and other epidemics are mentioned in general perspective. Afterward, the cell culture techniques used in viral vaccine bioprocesses are detailed, with an emphasis on vaccines which use the cell lines as expression platforms in their production. Then, selection criteria of the expression platfoms are detailed throughout the current regulations. Subsequently, the animal cell lines involved in production are introduced individually. Here, some cell lines used in commercial production for the first time in new vaccine types that have been put into clinical practice have been introduced as “vaccine production platforms”. Detailed information is also given for each cell line with vaccines which they involve as the expression platform. Also, candidate vaccines that are being developed using these cell lines are also briefly mentioned. Finally, other potential cell lines, which are not currently used in commercial production, are considered as new expression platforms by referring to their preclinical studies and the future of cell-culture based vaccines are discussed in detail.

## 2 Currently Available Viral Vaccine Types

Vaccine discovery, production, and development is a process that dates back thousands of years by different practices. However, the development of approved vaccines in a scientific manner began with Edward Jenner and has been ongoing for almost 250 years.^2,3^ Over time, many vaccines have been developed against various diseases. While some of these failed, such as the typhus or acquired immunodeficiency syndrome (AIDS) vaccine, the clinical use of successful ones still continues. The success of vaccines is basically evaluated on two parameters, safety and effectiveness.^19,22^ In terms of safety, it is desired that there are no or very few side effects after the administration of a vaccine. Especially when evaluating these side effects, in addition to the reactions that develop primarily due to reactogenicity and immune response, the secondary effects that occur on the whole body immediately after the administration or in the long term should also be examined in clinical studies.^4,20,23^ Effectiveness can be defined as the success of vaccines in protecting the individual against the relevant disease for which they were developed. This is possible by detecting and processing the antigenic part or parts of the relevant pathogen in the vaccine by the immune system of the applied organism. For this, it is necessary to determine the antigenic parts of the pathogen and define them correctly in terms of structure. Afterward, the antigen (or antigens) selected as the main component that will stimulate the immune system for production must be synthesized correctly and this form must be maintained in the vaccine formulation.^1,8,19^

Vaccine development is a very difficult process. Because it involves many different time-consuming scientific steps which requires multidisciplinary work by molecular biologists, biochemists, genetic engineers, bioengineers, pharmacists, and medical experts. Especially, in preclinical studies the vaccine’s fundamental features, uptake pathways, ability for immune stimulation, and formulation are determined by in vitro and in vivo studies. After that, clinical studies take place and finding appropriate healthy volunteers can be very challenging, as seen in the Covid-19 pandemic. Also, in all of these steps safety regulations and required authorization documents must be provided to be commercialized. However, even if the vaccine is approved, while the commercial bulk production takes place, the safety and efficacy of each batch have to be guaranteed according to the safety regulations.^24−27^

The primary step in the development of a vaccine is the correct identification of the pathogen causing the disease.^4,19^ This step is very vital and provides details of what the disease agent is, where it originates, how the disease occurs, how it affects the body, and the proliferation mechanism of the disease agent.^18−20^ Additionally, if this disease agent has newly emerged, it must be determined which host it came from, how it can be transmitted to humans, or how it has undergone mutation and acquired different properties. The pathogen identification step facilitated by the technologies and knowledge that have developed over time. In this respect, especially considering Covid-19 and influenza diseases, it is important to reveal how these viral agents differ from their original ancestral virus structures and how they become more contagious or lethal. In this way, the accuracy in creating the antigenic immune response, which forms the basis of the vaccines to be produced, will be at the highest level.^1,9,28^ In this respect, the development of microbiology and genetics and the distinction between bacteria and viruses have moved the science of vaccines forward.^3^

Bacterial vaccines generally focus on directly multiplying the microorganism that causes the disease, and since most of the bacteria have the biomechanism to directly multiply themselves, their production process can be carried out more easily in bioprocess perspective. Because the bacterial pathogen itself does not require any expression system. Therefore, using basic culture media (when compared to animal cell line cultivation medium contents) and under specific production conditions, many bacterial vaccines can be produced. In this process, the species selected for vaccine production is propagated in suitable nutrient media^1,19^ and then downstream processes are carried out depending on the type of vaccine desired to be produced. However, the development and production of bacterial vaccines is also a challenging process in terms of clinical studies, control of biosafety in the entire production bioprocess, and meeting the requirements of regulations and quality regulations.^9,29^

**Figure 1:**
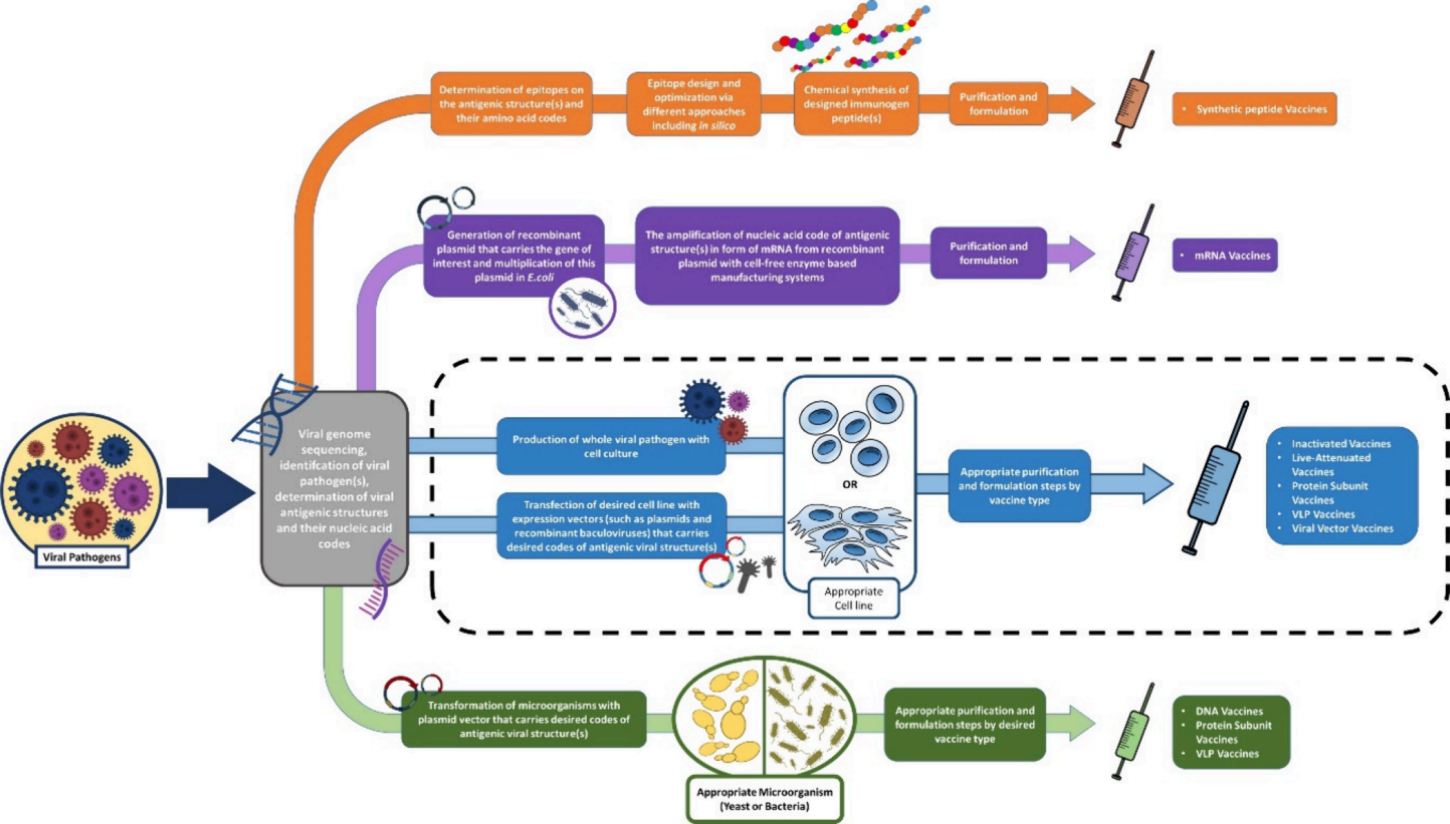
Up-to-date viral vaccine types. Orange line, synthetic peptide; purple line, nucleic acid-based; blue lines, animal cell culture-based; and green line, microorganism-based vaccines.

In vaccines developed against viral pathogens, the vaccine production process occurs indirectly due to the nature of the virus. Here, a live expression platform is needed to accurately synthesize the relevant virus or the relevant antigenic region selected as a vaccine candidate (Figure 1).^9,17^ Different vaccine types and appropriate production strategies have been adopted to ensure effective immunity. Traditional vaccine types that have been produced commercially for years; these are inactivated, attenuated, and subunit vaccines. The approval and commercialization processes of newer approaches, such as mRNA, DNA, virus-like particle (VLP), and viral vector vaccines (Table 1), have accelerated, especially with epidemics such as Ebola and Zika and with the Covid-19 pandemic.^1,7,20,21,29^

**Table 1:** Comparison of Viral Vaccine Platforms Pandemic Response Speed by Emphasis on Their Advantages and Drawbacks

viral vaccine type	expression platform	advantages	drawbacks	pandemic response speed
inactivated vaccines	cell line	• no risk of virulence reversal	• limited cell-mediated immunity	***
		• sufficient clinical history record	• variable efficacy	
		• good vaccine safety profile	• seed virus need	
		• relatively strong and broad immune response	• requirement of appropriate level biosafety facilities to grow live virus	
		• stable in storage and easy distribution	• potential epitope alteration by inactivation step	
		• relatively fast and scalable manufacturing by well-known bioprocess technology	• require multiple administration dose	
			• risk of unwanted contaminants	
			• lower vaccine purity profile	
			• not all viruses adequately propagated in cell lines	
live attenuated vaccines	cell line	• inducing very strong immune response in terms of both humoral and cell-mediated immunity	• risk of regainig virulence	*
		• native viral antigenic structure(s)	• required long time for developing attenuated vaccine candidate virus	
		• mimic natural infection	• risk of disease for immunocompromised patients	
		• long-lasting protection	• requirement of cold chain for storage	
		• less administration dose requirement	• more higher risk in production process	
		• well-established tehnology with sufficient clinical data	• seed virus need	
			• requirement of appropriate level biosafety facilities to grow live virus	
			• not all viruses adequately propagated in cell lines	
subunit vaccines	cell line, microorganism	• noninfectious vaccine content	• poor immunogenicity without adjuvant or conjugation	**
		• higher safety and fewer side effects	• limited induction of cell-mediated immunity	
		• more immunogenic antigen(s) can be chosen specifically	• risk of wrong antigenic conformation especially production via microorganism-based expression platfom	
		• well-known technique and manufacturing	• need of higher titer antigen	
		• high stability and ease in storage	• directed immune response to only relevant target antigen	
			• complexity of vaccine development and manufacturing process	
			• require multiple administration dose	
virus-like particle (VLP) vaccines	cell line, microorganism	• noninfectious vaccine content	• limited immunogenicity and can require adjuvant	**
		• broad antigenic profile and dense epitope	• high overall bioprocess cost	
		• native whole virus conformation and can mimic natural cell-entry	• very complex vaccine development and manufacturing process	
			• low vaccine purity profile	
			• lower stability and need of special conditions for storage	
viral vector vaccines	cell line	• less infectious and safer than attenuated vaccines	• pre-existing immunity can cause lower vaccine efficiency	***
		• native viral antigenic structure(s) and any antigen can be targeted specifically	• higher risk for adverse reactions	
		• mimic natural infection	• very complex vaccine development and manufacturing process	
		• manufacturing can be established without seed virus stock	• high cost and limitation in scale-up	
		• very strong immune response can be induced in both cellular and humoral	• replicating vector vaccines are not suitable for immunocomprised patients	
		• for replicating viral vectors lower or single dose can be sufficient	• risk of genomic integration	
		• fast and scalable manufacturing process	• dominantly induce cell-mediated immunity and lower humoral immune response	
			• require very low storage temperature	
nucleic acid based (DNA and mRNA) vaccines	microorganism	• noninfectious vaccine content	• poor immunogenicity	****
		• very fast and relatively cheap manufacturing	• need for special delivery systems/devices and appropriate vaccine formulation	
		• especially DNA vaccines are very stable	• especially mRNA platform is very unstable and extremely low temperature cold chain is nedeed	
		• good safety profile	• risk for genomic integration in DNA vaccine platform	
		• high purity	• very new platform there in no sufficient clinical data and record history	
		• native antigenic structure	• difficult cell-entry	
		• no risk for genome integration in mRNA vaccine platform	• risk of unwanted RNA induced interferon response	
			• adaptable platform for new pathogens	
synthetic peptide vaccines	chemical synthesis	• excellent purity and safety	• very poor immunogenicity	****
		• noninfectious vaccine content	• requirement for adjuvant and conjugation	
		• nondependent with biological expression system	• peptides are very unstable and prone to enzymatic degradation	
		• scalable, cheap and rapid production	• inducing immune system is very hard for single peptides	
		• minimal adverse reactions	• no currently licensed vaccine using this platform	

### 2.1 Inactivated Vaccines

Inactivated vaccines are the most commercially used and well-known vaccine type in terms of production technology. These vaccines are basically produced by propagating the viral pathogen with cell culture technology and then inactivating the produced bulk virus by chemical (formalin, β-propiolacton, ascorbic acid, etc.) or physical (temperature, pH, gamma irradiation, etc.) methods.^1,17,20^ Since it usually contains the inactive form of the entire viral agent or the fragmented entire viral agent, it can create a broad-spectrum immune response.^17,20,21,28^ Since it contains only inactive viral pathogens, it is a very safe vaccine type and is easy to store. In addition, it is a very suitable vaccine type for relatively rapid viral vaccine production following the identification of the virus in a scenario such as a pandemic. Also, since it has been the most preferred vaccine type for many years, there is high knowledge about this technology and the general production cost is low.^1,7,8,30^ For this reason, inactivated vaccines have become applicable to all countries of the world, regardless of their level of development. Although this type of vaccine can trigger humoral immune response, it cannot induce a strong cell-mediated immune response.^6,17,31,32^ Because the success rate of mimicking natural infection is quite low for inactivated vaccines. In addition, since they have low immunogenicity as a result of inactivating the entire viral agent, repeated administration, or higher doses are needed to create and maintain an adequate immune response that will provide protection from the relevant viral disease.^8,20^ Adjuvants can be added in inactivated vaccine formulations to overcome this problem. There are commercially developed inactivated vaccines against many viral diseases such as influenza, rabies, polio, and hepatitis A.^1,6,8,17,21^

### 2.2 Live Attenuated Vaccines

Attenuated vaccines are developed by serial passaging of wild-type viral pathogens in cell cultures/eggs at high rates and under special environmental conditions. Thus, viruses separated from human physiological conditions and the immune system evolve to exhibit greatly reduced virulence compared to their wild types.^1,6,14,20^ While this selected evolution is taking place, it is not desired that they completely lose their ability to infect the target organism and multiply. Thus, when these attenuated viral pathogens are later applied to the target organism as a vaccine, they can successfully mimic natural infection by multiplying without causing a serious disease.^1,6,7^ Thanks to these abilities, attenuated vaccines can stimulate both humoral and cellular immunity to a high extent by activating all biological defense mechanisms as in real infection without any need of adjuvants. However, since they still contain the entire live viral pathogen, their safety needs to be confirmed.^20,21^ The possibility of gaining high virulence properties again, especially with potential mutations that may occur, is a very high risk for this vaccine type.^17,23^ The necessity of developing a viral strain with reduced pathogenicity and proving its safety, which is the basic step in the production of this vaccine, is both time-consuming and costly.^1,17^ Although molecular biology methods such as codon-deoptimization have been preferred in recent years to prevent these disadvantages for faster strain development, these processes can still be quite time-consuming, unpredictable, and complex.^1,32,33^ In addition to all these, cell culture technology is used in the production of attenuated vaccines, as in inactive vaccines, since the first step is the production of the viral agent.^15,20^ However, unlike inactive vaccines, here the attenuated viruses takes place in the vaccine formulation without being inactivated.^8,19^ Commercially attenuated vaccines have been used for many years against viral diseases such as measles, chickenpox and rubella.^1,7,21^

### 2.3 Subunit Vaccines

Subunit vaccines are vaccines that do not contain the entire virus and contain only a certain antigenic part or a fragment of the antigenic part found in the structure of the relevant viral pathogen. For the production, the focus is on obtaining only the part that shows antigenic properties.^1,7,9,21^ In this respect, the production of subunit vaccines is carried out in two ways. The first of these is to produce the actual viral pathogen and then purify this antigenic part or parts from the entire virus produced. FLUAD, a subunit adjuvanted influenza vaccine, can be given as an example. In the production of this vaccine, the entire virus is first produced and after it is inactivated, the desired antigenic parts, hemeagglutinin, and neuraminidase, which is contained in a much smaller amount in the vaccine, are purified.^28,32,34^ The second is the use of recombinant DNA technique, which allows the synthesis of only this antigenic part. Vaccines produced by this method are generally called recombinant subunit vaccines.^17,28,35^ This second method is more preferred. Because, thanks to this technology, only the antigenic part (or parts) of interest is synthesized instead of the entire virus, and it is a lower-cost, faster, and safer method in terms of the overall bioprocess flow.^1,8,9,36^ Subunit vaccines produced by both methods are very safe in terms of clinical application and side effects. However, although their safety is high, similar to inactivated vaccines, subunit vaccines trigger more of a humoral immune response and create a low cellular immune response. Since this antigenic portion produced in subunit vaccines is highly purified and very small in size compared to the entire virus, they should be used with immunostimulators such as adjuvants or carrier systems to be seen by the immune system and stimulate the appropriate immune response.^1,20,28,36^

The most important step in the production of recombinant subunit vaccines is choosing the appropriate expression platform that can be successfully transfected and subsequently synthesize the relevant antigen in the correct conformational form.^1,35,36^ When appropriate post-translational modifications (PTM) do not take place, it may be possible that no immune response is induced by the immune system or a weak immune response may developed. In this situation the antigenic structures of the virus in natural infection does not fully match for adequate protection. For this reason, yeasts and animal cell cultures, which are eukaryotic systems that can perform appropriate PTMs, are preferred.^1,9,18,35^ The subunit vaccine platform was first commercialized with using recombinant DNA technology in 1986 with the vaccine developed against Hepatitis B virus, which is very difficult to produce,^37^ in yeast cell culture.^38^ Subsequently, this platform has become the preferred system not only for the production of the hepatitis B vaccine but also for the production of other approved commercial viral vaccines developed against influenza, shingles and, most recently Covid-19.^28,35,36^

### 2.4 Virus-Like Particle (VLP) Vaccines

Virus-like particles (VLP) are particles that are similar to the original viruses in terms of morphological and antigenic properties, but do not have the ability to replicate because they do not have viral genome.^39,40^ The VLP platform is similar to inactive vaccines in terms of safety. On the other hand, this platform is similar to subunit vaccines in terms of its production process, as it can be synthesized with or without an envelope by many different expression systems such as bacterial, yeast, plant, or animal cell cultures.^1,7,20^ In this respect, it is necessary to first create the plasmid or recombinant baculovirus encoding the VLP designed to be used as a vaccine, taking into account the expression system desired to be used in production.^39^ The most important parameter in VLP design is that the antigenic and geometric structures of the original virus can be synthesized correctly and self-assembled in the selected expression system.^1,39^ For this, the plasmids (or recombinant baculoviruses) created for VLP production must contain gene cassettes that provide these features in the genome of the original virus. The immunogenic properties of the produced VLPs after/during production can be increased by different modifications such as peptide conjugation, chemical cross-linking, or adjuvant addition.^39−41^

VLP vaccines have been shown to be much more effective than subunit vaccines, which are a similar technology, in terms of stimulating the immune system.^39^ VLPs mimic infection more successfully in terms of cellular uptake, and since they contain many different viral antigenic structures, they can create a high level of antibody response against different epitopes and also trigger cellular immunity.^1,7,39−41^ However, the disadvantages of this system are the complexity in its production, high production cost, stability problems, and the difficulty of creating a VLP structure.^39,40^ Nevertheless, due to the superior advantages it provides, the VLP vaccine platform has become a highly preferred technology, especially among vaccine candidates developed or under development against Covid-19.^21,39,40^ In this respect, there are many VLP Covid-19 vaccine candidates in approval process and among these, only plant-based Covifenz has been approved for use in Canada.^20,42^ Additionally, there are currently commercial VLP vaccines developed against human papillomavirus (HPV) and hepatitis B virus.^39−41^

### 2.5 Viral Vector Vaccines

Compared to other viral vaccine platforms, antigenic region or regions in viral vector vaccines are not directly included in the vaccine formulation. Here, carrier viruses called viral vectors are used for immunization.^1,24^ In this respect, viral vector vaccines are a platform that uses recombinant viruses designed to have the genetic code of specific antigenic structure or structures of relevant viral pathogen that causes disease.^1,20,21^ Thanks to the infection with using a viral vector other than the original disease-causing viral agent, the genetic code of the desired antigen to be expressed is carried to the body cells in the immunization area. In this way, only the antigenic part(s) of the actual viral pathogen, without the presence of it, are synthesized with a very high conformational accuracy by the receiver’s own cell-machinery system, and the immune system is stimulated in this way; thus, as if a natural infection takes place in the body with the pathogen.^1,7,24^ Since the antigenic regions that will stimulate the immune system are endogenously synthesized by the cells themselves, they can stimulate both humoral and cellular immunity at a high level.^20,24,43,44^ In addition, the other advantages of this platform are that it provides high-fidelity gene transfer and expression, rapid production on a large scale, and the convenience it provides in terms of vaccine storage conditions.^1,7,20,24^

Viral vector vaccines are divided into two types: replication deficient or replication competent (self-amplifying).^45^ Although vaccines that can stimulate the immune system well even at very low doses can be developed with replication competent viral vectors, there are safety concerns due to these properties.^1,20,24,45^ These are similar to attenuated vaccines in this sense.^1,24^ Replication-deficient viral vector vaccines are an improved version of this platform technology, and the carrier viruses used here transfer the relevant genetic code to the cells in the immunization areas without replicating themselves. In this respect, replication-deficient viral vector vaccines are safer, but since the viruses used cannot replicate themselves, they must be prepared in higher titers in vaccine formulations.^1,24,45^

Many different types of viruses are used in viral vector vaccines, depending on the system designed. These include engineered viruses such as adenoviruses, adeno-associated viruses, alphaviruses, herpes viruses, poxviruses, measles viruses, vesicular stomatitis virus, rabies virus, influenza viruses, parainfluenza virus, parvoviruses, and lentivirus.^24,44,46,47^ However, before these viruses are used as viral vectors, they are made safer by changing their pathogenicity, infectivity, and replication properties compared to their wild type, according to the vaccine design.^24,44^ Although different viral vectors are used in vaccine development in terms of replicative properties and virus types, the common feature of all of them is they do not cause disease in humans and do not integrate into the host genome.^1,24,43,45^ Viral vector vaccines, whose commercialization process was accelerated due to the epidemics that occurred before the Covid-19 pandemic, were approved for use against Japanese encephalitis and Ebola. These approved vaccines are replication competent. However, with the Covid-19 pandemic, replication-deficient viral vector vaccines were introduced to the market for the first time.^1,7,20,24,44^

### 2.6 Nucleic Acid Based (DNA and mRNA) Vaccines

Although their therapeutic usability has been evaluated for many years, another vaccine type that entered clinical use for the first time due to Covid-19 is DNA and mRNA vaccines. These vaccines are created simply by using the genetic code of the antigenic region of the relevant viral agent as DNA or directly as mRNA in vaccine formulations.^1,20,25,48^ Here, naked DNA or mRNA carrier is given directly to the cell via a vesicular system, and the immune system is stimulated as a result of the synthesis of these antigenic region or regions using natural cell-machinery, as in viral vector vaccines.^7,48,49^ Studies have shown that these vaccines can stimulate both cellular and humoral immunity, and that the process of mimicking infection, which is the main target of all vaccine types, occurs in a very short time.^1,49−51^ In addition, they are very advantageous systems in terms of cost, as they can be developed very quickly and plasmid DNA can be obtained at high efficiency with only simple bacterial systems.^1,49,51^

In particular, the production process of mRNA vaccines, which can be synthesized with cell-free enzymatic systems by using only a certain amount of plasmid as raw material, stands out in terms of both cost and speed by keeping dependence on any expression system at minimum.^25,48−50^ In addition, DNA vaccines are more advantageous in cold chain and storage compared to mRNA vaccines because they have a more stable structure.^1,20^ However, there are drawbacks or unknowns such as lacking of clinical data to evaluate the long-term side effects of these platforms, their interaction with the receptors in the cell after cellular uptake, the need to evaluate in more detail whether they cause undesirable damage while providing antigen presentation in the cell, the need to evaluate other mechanisms during cellular transcription and translation, whether it has an undesirable interaction with cellular systems, the potential for misexpression and the resulting waste protein accumulation.^48−52^

Today, mRNA vaccines with the trade names COMIRNATY and SpikeVax to be used for Covid-19 have been applied to humans for the first time with emergency use approval, as the precursors of this platform technology.^1,20^ In addition, different mRNA vaccines with the same platform technology have recently received emergency use approval and have been implemented. In addition, a single plasmid DNA vaccine called ZyCoV-D has been approved for emergency use in India and has been put into clinical practice.^21,53,54^

### 2.7 Synthetic Peptide Vaccines

Synthetic peptide vaccines, like nucleic acid-based vaccines, are a type of vaccine that have been implemented for the first time, with their commercialization accelerating with the Covid-19 pandemic. Synthetic peptide vaccines are an approach developed by focusing on the epitope regions of the antigenic viral parts that enable the development of an immune response against the virus, to which the immune system cells respond.^1,55−57^ Epitopes can be defined as special amino acid sequences located on the relevant viral antigen, which can be seen by the immune system and develop both an antibody response and a cellular response after being processed by antigen-presenting cells.^1,55,58,59^ With this approach, it is thought that giving only the relevant epitope region or regions to the person in peptide form, instead of using the entire antigen in the vaccine, will provide adequate immunization.^27,58,60^ Therefore, for vaccine production, the amino acid code of the epitope is first determined in order to synthesize the epitopes on the relevant antigen in peptide form, which stimulates the immune system and creates an immune response against it.^56,60,61^ In determining these codes meticulously, in silico methods are used to design epitopes, taking into account parameters such as immunodominance, conformational structure, in vivo stability and degradation, and cellular uptake. This designed epitope is then synthesized chemically in the form of a synthetic peptide without using any expression system. The length of these immunogenic peptides synthesized is generally in the range of 20–30 amino acids.^57,60−62^

The biggest drawback of this approach is that the synthesized peptides have low immunogenicity because they are very pure and small molecules.^57,59,60^ Therefore, synthetic peptides must be used together with immunomodulators like adjuvants. In addition, since these peptides are unstable and rapidly degraded in in vivo systems due to their own structure, they require conjugation with other molecules or appropriate carrier systems.^57,61^ In addition, synthetic peptide vaccines has advantages such as can be synthesized in a completely chemically defined manner, are water-soluble, maintain a stable structure under storage conditions, do not require any live expression system,^1,57^ can be produced on a large scale easily, have minimal side effects (like allergy) with carrier system optimization, and when they compared with conventional systems production can be done at a very low cost.^56,60,61^ EpiVacCorona synthetic peptide vaccine developed against Covid-19, which has received emergency use approval for use only in Russia and a few countries, and its effectiveness has been demonstrated by comparison with other vaccine types.^58,63^ In addition, synthetic peptide vaccines are also being developed against viral agents such as influenza, HIV (human immunodeficiency virus), hepatitis B, and HPV.^55,56^

## 3 Use of Cell Lines in Vaccine Production

It is not possible for viruses to multiply without living systems thus, sick people and live animals were initially used in vaccine production. It is known that in addition to the use of cattle and horses in the production of vaccines developed by Jenner against smallpox, a human-to-human vaccination method was also used.^2^ Over time, with the development of the smallpox vaccine production method and Pasteur’s discovery of the rabies vaccine, vaccine production was carried out entirely on live animals.^2,3^ However, this situation has brought about many problems in terms of both vaccine quality and reliability and ethics. The first important step in preventing these problems was in 1931 when Ernest William Goodpasture and his team showed that viruses could be propagated in embryonated chicken eggs.^11^ At the same time, egg-based vaccine production has disadvantages such as the constant need for specific pathogen-free (SPF) eggs, the need for at least 1–2 eggs for each dose of vaccine production, the inability to produce sufficient titers of some viruses, the adaptation can required for some viruses to multiply in eggs, potential side effects in individuals with egg allergy, intense workload requirement, and high production costs.^14,64^ However, egg-based vaccine production is still taking place today. The reason for this is the production processes that can be considered as traditional and as a result of their successful use for many years and the cooperation of companies producing egg-based vaccines and institutions producing SPF eggs. Today, some of the vaccines produced against influenza^17,65,66^ and yellow fever are egg-based.^67,68^

In the years when virus production experiments were carried out in eggs, the concept of in vitro cell culture began to develop by making great strides. Especially in those years, while the cell culture technique was mostly known for studies on how to preserve cells outside the body without deteriorating their structural properties, it has made significant progress in a short time with the development of culture media formulations, the discovery of antibiotics and their use in media contents, the development of aseptic techniques, and the identification of growth factors.^3,13,69−71^ As a result of the development of the cell culture technique and the demonstration of its continuous availability, this technique has come to the fore for the production of viruses whose replication depends on living systems.^6,13,15,17^ With the Nobel Prize winning study conducted by Enders, Robbins, and Weller in 1949, in which it was shown that the polio virus could be produced in cell culture, the industry is focused on cell culture technique for vaccine production.^72^ Jonas Salk was the first to use cell culture in vaccine production with the polio vaccine he developed in 1954. Salk produced this vaccine in primary monkey kidney cells.^3,4^

Chicken embryonic fibroblast (CEF) culture, especially obtained from embryonic chicken eggs, is the most commonly used primary culture in vaccine production. This CEF culture, which is still used in vaccine production today, is considered the gold standard in almost all vaccine studies and especially in attenuation studies.^4,12,15,23^ However, these cultures have drawbacks such as the need to sacrifice a great number of livings due to the need to be reobtained in each process cycle, problems in cell banking, very short passage life, uncertain cell characters, and the presence of potential contaminants from the organism from which they were obtained.^4,12,23^ Simultaneously with the increasing world population over time, the need for more reliable, effective, cheap, short-term production, and large quantities of vaccines has emerged. In order to meet this need and to eliminate the disadvantages of primary cultures, various cell lines have been developed and started to be used for vaccine production.^6,12,13,17,72^

Cell lines were developed to eliminate the disadvantages of primary cultures, which are difficult to characterize and have constantly variable culture structure. Another reason for searching for a production platform other than primary cultures was the discovery that many polio vaccines produced with primary monkey kidney cells between 1955 and 1963 were actually infected with simian virus 40 (SV40).^10,72^ Therefore, in order to prevent such a situation from recurring, efforts have been made to create cell cultures that have been fully characterized for safety. First of all, diploid cell lines (or diploid cell strains) have been developed, which can actually be considered as primary cultures because they are created by passage of primary cultures.^4,12,13,17^ Later, continuous cell lines were developed to overcome the disadvantages of diploid cell lines, such as limited passage number, sensitive structure, and dependence on complex nutrient medium components. Continuous cell lines are defined as cells that are stable, can divide continuously, and can be cultured indefinitely in vitro under appropriate conditions.^12,69,71^ However, at first, the fact that continuous cell lines were “immortal” led to thoughts that their use in production would be unsafe.^10,12,73^ It took a long time for them to be used as cellular expression platforms in vaccine production, both because the techniques that would prove that their use was safe were insufficient at that time and because of the lack of studies on virus production on this subject.^4,6,10,12,23^ It has been shown that continuous cell lines can be used in vaccine production after developing technology and determining the necessary rules to eliminate potential risks and obtaining all approvals.^4,5,69^

## 4 Selection of the Expression Platform

The basis of all commercial applications based on cell culture focuses on the production of high amounts of cells to be used as an expression platform or for direct treatment in the field of personalized medicine.^9,29,74,75^ The reason for this is that there is a direct proportion between the amounts of end products such as biomass, monoclonal antibodies (mAb), growth factor, hormone, and viral vaccine that are desired to be obtained at the end of these productions and the total number of cells that can be reached during production.^9,13,29,76−78^

### 4.1 Production Steps and Overall Vaccine Bioprocess

**Figure 2:**
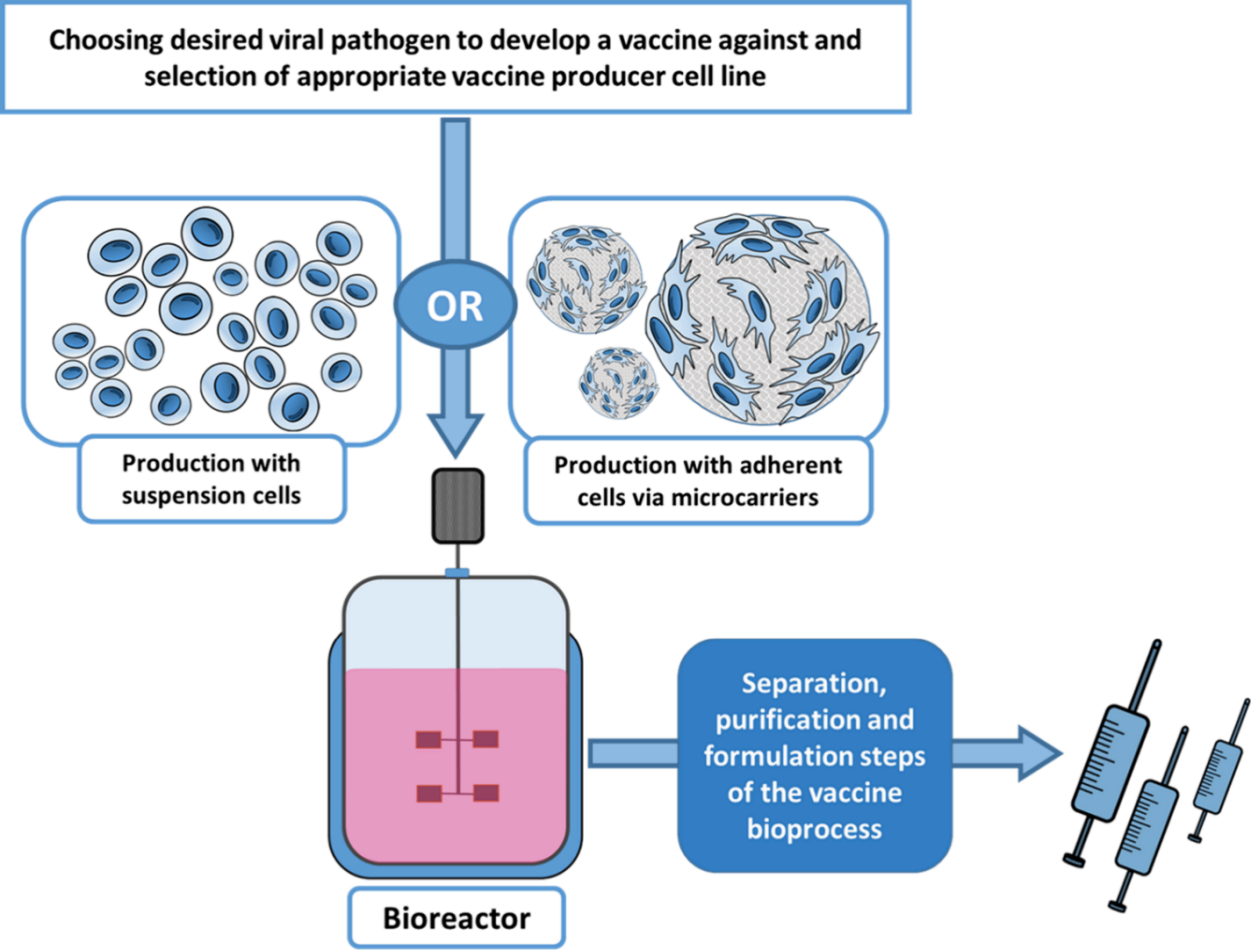
General bioprocess of animal cell-culture based viral vaccine production.

In cell culture-based viral vaccine production, after determining the type of vaccine and production technology to be produced, the virus, viral antigen(s), or viral vector to be used as vaccine material must first be produced. Therefore, the expression platform that will produce them, that is, the cells, needs to be proliferated.^9,69,74,79,80^ Although these productions can be carried out with static cell culture systems on a small scale, bioreactors are used due to the superior advantages they provide, especially in industrial productions where production takes place in large volumes.^17,74,75^ Bioreactors are particularly suitable systems for scale-up, and thanks to their high controllability and automation features, they stand out in ensuring the necessary safety regulations and GMP conditions in vaccine production (Figure 2). In the production of viral vaccines, production is carried out in large-scale bioreactors of different types and configurations, depending on the production feature of the chosen expression system. Selection of the expression platform, development of this expression platform if necessary (use of molecular genetic techniques such as recombinant DNA and adaptation to production conditions or the nutrient medium to be used in production, etc.), culture medium and inoculation preparation, optimization of process parameters, and production in bioreactors are all named upstream processes.^9,29,69,74^ Following this, downstream processes are carried out specifically for the type of virus planned to be produced and the type of vaccine targeted. Especially in processes based on whole virus production, viruses to be used as vaccine materials are generally released directly into the production environment by budding or lysis of the infected cell. However, this situation varies depending on the type of virus to be used in production.^9,29^ In this regard, in some cases, in order to successfully obtain the virus, cells must be disrupted mechanically or by chemical methods using surfactants. Particularly in vaccines where direct production of the entire virus is not required, many of the downstream processes in the production processes include the lysis step of cells. Examples of vaccine types that require this are subunit, viral vector (especially adenovirus-based ones) and VLP vaccines.^17,29,69,81^ In this way, after obtaining the virus or viral antigen to be used as vaccine material, the concentration step and, depending on the type of viral vaccine, advanced purification steps such as inactivation, filtration, and chromatography constitute the downstream processes.^9,17,29^

### 4.2 Cell Line Selection

The selection of the appropriate expression platform to be used for vaccine production is the most important stage of the entire production process. The reason for this is that the available cellular substrates that will synthesize the desired component as a vaccine material in sufficient quantities and in the correct structure to have the appropriate immunogenicity have very different properties. If the vaccine component in the targeted production does not match the features of the selected platform, then successful production cannot be achieved.^9,19,69^ Especially considering the type of vaccine desired to be produced, cellular substrates with appropriate properties that can be infected with the relevant virus and produce the virus or can correctly synthesize the viral antigen(s) to be used as vaccine material with genetic engineering must be selected.^17,72,82^ Additionally, there is a direct approach for the expression platforms used to produce human vaccines should be of human origin. In this sense, some studies show that the vaccine material produced using human cell substrates expresses the virus in higher amounts and in more accurate forms in terms of viral specificity, since it is synthesized in the original virus-host relationship, and therefore provides more effective immunization.^5,15,16,18^ Along with these, after the selection of the appropriate cell line, the sublines derived from this cell line should also be evaluated. Some sublines may be more suitable or efficient in terms of intended production than the selected ancestral cell line.^16,83^ For instance, the Vero.E6 subline derived from the Vero cell line used in the production of inactive Covid-19 vaccines allows the production of higher viral titers.^83^

The growth character of almost all of the cell lines used in cell culture-based vaccine production is adherent.^80^ Since adherent cultures need suitable surfaces for growth, scale-up requires the use of support materials such as microcarriers (Figure 2).^74,84^ This situation creates additional costs in production and prolongs the preprocessing time in preparation for production. Additionally, it is difficult to obtain large amounts of cells because cell proliferation is limited by the available surface area.^69,74,85,86^ Since it is known that primary cultures and diploid cell lines retain their adherent character and have limited adaptability to suspended conditions, they are produced adherently in viral vaccine production, either using microcarriers or in packed-bed bioreactors (such as iCellis) where other suitable support materials are used.^69,80^ Cells with suspension character have advantages such as ease of scale-up since they are not dependent on any surface, less labor required, growth is limited only by the cell concentration in the nutrient medium, and the cellular microenvironment in the production environment is more homogeneous.^69,87^ However, as mentioned, since almost all of the expressional platforms used in vaccine production show adherent character, if the use of suspension cultures is aimed in production, the cellular substrates must be adapted to the suspension growth condition.^69,86^ This adaptation is possible by culturing adherent cells in dynamic systems, using serum-free nutrient media specifically developed for the cell line, or applying both methods simultaneously.^69,86,88,89^ In this way, very high success can be achieved in continuous cell lines.^4,12,13^ However, since the cellular character changes greatly during adaptation, it must be constantly checked that this suspended culture developed by cell engineering synthesizes the vaccine material in sufficient quantities and in a suitable form for industrial use. Additionally, the safety properties of adapted cells, such as tumorigenicity, should also be re-evaluated and checked.^23,69,88^ The main disadvantage of suspension cultures is the need for cell-retention devices in case of any nutrient media change or washing process during production.^69^

### 4.3 Use of Serum-Free Medium

**Figure 3:**
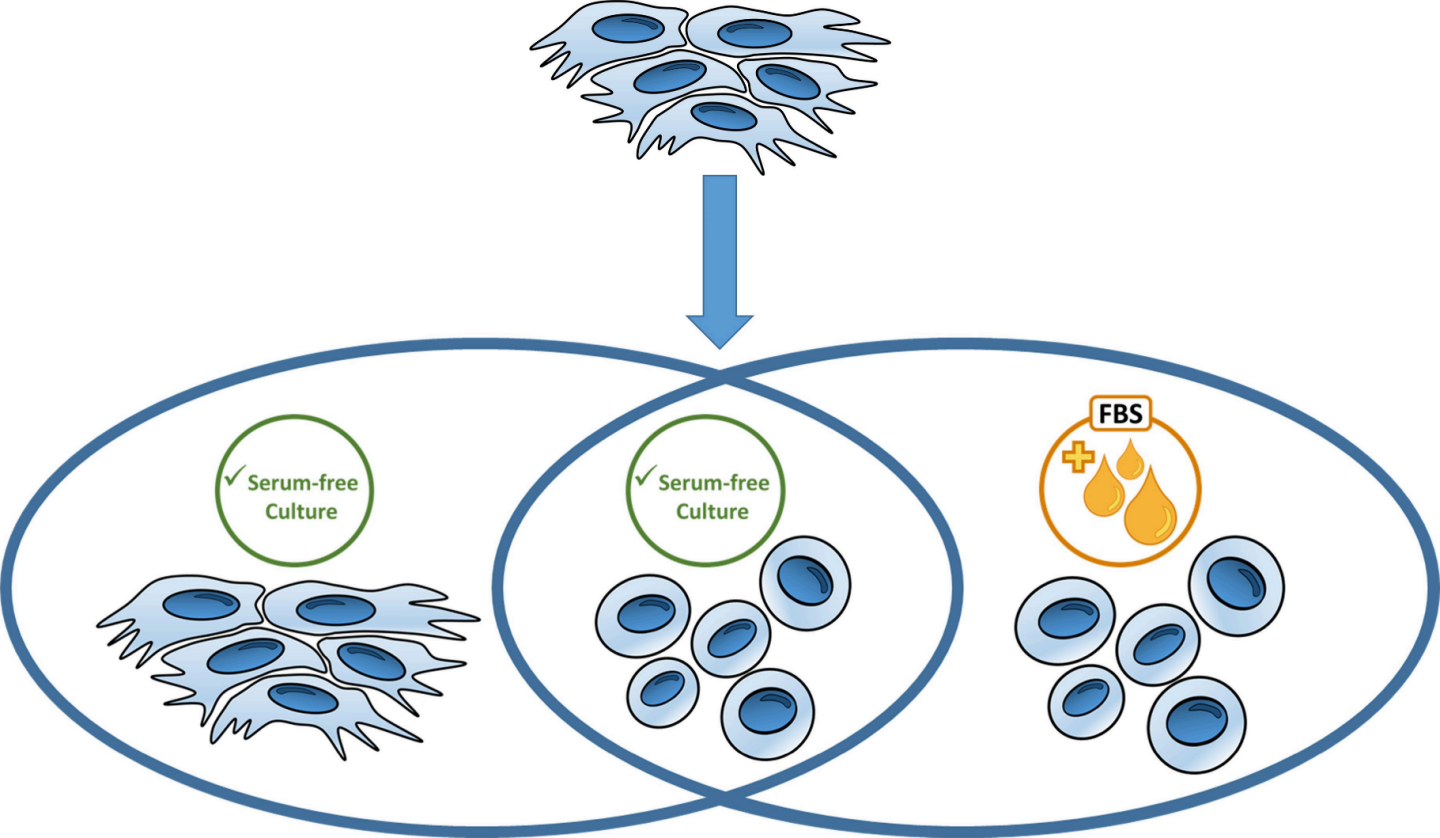
Adherent cell lines can be adapted to serum-free culture conditions, suspension growth or both of them.

In recent years, there has been a trend toward the use of serum-free medium in cell culture-based industrial production. This is because in the guidelines published by authority organizations such as the American Food and Drug Administration (FDA), the International Council for Harmonization (ICH), and the European Medicines Agency (EMA), the use of serum is not recommended in the processes where biotechnological product production is carried out for use in human health.^88−90^ It has been shown that completely eliminating or reducing the use of serum in production is advantageous in terms of cost, ease in downstream processes and individuals who are allergic to animal originated products.^90−92^ Serum-free media are also used, as mentioned, to adapt adherent cultures to suspended growth conditions^69,86,88^ (Figure 3). However, the need for complex culture media components, especially for primary cultures and diploid cell lines, makes it necessary to culture them in richer composition.^93^ For this reason, viral vaccine production based on all virus production, especially where cellular substrates such as primary culture and diploid cell lines are used, is carried out in two stages in order to reach maximum cell concentration quickly and to eliminate the disadvantages of serum use. For this, first, the cells are cultured in serum-containing medium to reach the maximum concentration in the culture medium, and then this medium is replaced with serum-free virus production medium.^17,29,74,80^ It provides a much higher success rate in the adaptation of continuous cell lines to a serum-free medium compared to other cellular substrates.^12,18^ Since continuous cell lines can be produced with similar or better proliferation performance than they show in serum containing media, only serum-free culture media can be used throughout the viral vaccine production process without any shift requirement.^69,74,94^ Today, many different brands of serum-free media are available for continuous cell lines used in the commercial production of many different biopharmaceuticals, such as Chinese hamster ovary cell (CHO) and African green monkey kidney cell (Vero).^82,89,93^

### 4.4 Safety Regulations

In cell culture-based productions where cell substrates are used, many different parameters must be considered in terms of safety, apart from the composition of the culture medium. If sufficient documentation is not made to demonstrate the safety of the final product and the cell substrate on which this product is produced in accordance with the necessary regulations, then the relevant product cannot be commercialized as it will not be approved.^10,23,73,95^ For this, it is necessary to ensure the security rules determined by the authority organizations that approve the commercialization process. The most basic of these is that the expression platform used in production is not included in the final product, the viral vaccine, according to the standards in the pharmacopoeias.^4,12,23^ It has been shown that injections of continuous cell lines at high cellular concentrations induce tumor formation. This potential risk in production using these cells needs to be eliminated.^4,12,17,23^ Apart from this, another important safety regulation for viral vaccines is that it must be shown that the final product, the vaccine, does not contain any biological contaminants. This situation is very important in order to prevent the recurrence of this issue, which has become a public concern, especially after the administration of SV40-contaminated vaccines in history.^12,72^ Considering the serious side effects that may occur as a result of the possibility of a possible cell-originated pathogen being included in the final product during production, so it must be proven that the expression platform used in production is free from all adventitious agents.^96,97^ In this respect, the “cleanliness” of the cells obtained from approved culture collections should also be verified for their working cell bank in each production cycle.^6,12,18,80^ Another safety concern that arises specifically for continuous cell lines is that the end product of the production may trigger tumor formation. Because these cells often differentiated from the specific tissue from which they originate and become more similar to cancer cells. For this reason, it must be proven that, specific oncogenic viruses/the viral genome when creating a cell line (such as HEK-293) or from the cell line itself (such as HeLa), are not released into the culture.^5,13,17,23,95^ Compared to continuous cell lines, diploid cell lines stand out because they have been shown to be nontumorigenic and are very well characterized in terms of safety.^4,12,95,96^ Apart from this, in order to both trigger tumor formation and create an undesirable immunogenic response, the final product produced with all cellular substrates must not contain more than 10 ng of cellular genomic material^12,98,99^ and the DNA found must not be longer than 200 base pairs.^4^ In addition, even if this limit value is met, especially in production with continuous cell lines with a tumorigenic phenotype, the product must be shown to be safe by proving that the genomic material that may be present in the final product does not contain tumorigenic sequences through further molecular tests.^98,100^ It must be proven that, apart from tumorigenic nucleic acid sequences, different protein components or cellular factors secreted by cells into the culture medium do not have cancer-inducing properties. If it is known that such components are secreted into the environment by the cell, then it should be shown that they are removed by separation/purification processes and are not exist in the final product.^12,99,101^ The risk that may arise as a result of the presence of such potential tumorigenic components that may be secreted into the production environment varies depending on the tissue of origin of the cells used in production. In this sense, those with the lowest risk are epithelial and fibroblastic cells, while the cells with the highest risk are cells of hematopoietic origin. However, when risk assessment is made with advancing molecular techniques, all cell lines of different origins have the potential to be used in commercial production as expression platforms when their safety is proven.^10,12,95,101^ In this respect, cell lines that are not currently used as expression platforms in the biotechnology industry but have high potential in this regard should be evaluated.^102−104^

Another important criteria in ensuring the necessary safety regulations is the species barrier.^13,69,95,105^ The species barrier indicates that closely related species can be infected with the same viruses, while distant ones cannot be infected with these viruses.^96,101^ Increasing order of risk according to the origin of the cellular substrates used in viral vaccine production; avian and invertebrate cell lines, primate and nonhuman mammalian cell lines, primate cell lines, and human cell lines.^95^ While the species barrier provides an advantage in the production of other biopharmaceuticals and viral vaccines based on recombinant DNA technology, it poses an obstacle in vaccine production processes based on the production of the whole virus. Because, in vaccine production processes based on the production of the entire virus, the main product to be produced is the virus itself, which has pathogenic properties in humans. For this, the cell line desired to be used in production must be directly infected with this virus in order to produce.^6,19,69^ The use of human-derived cells, especially in processes where whole virus production is targeted, provides a natural advantage in terms of conformational accuracy and infectivity for the virus to be produced for use in vaccine content. However, it increases the risk of contamination with other potential viral contaminants in terms of species barriers.^4,16,18^ Another important parameter is viral susceptibility, that is, the ability of the cell to be infected with the virus. Although some cell types can be infected with different viruses, the virus may not be produced to meet commercial expectations.^6,8,14,17^ For this reason, the cellular platform to be used must be able to both infect the virus desired to be produced and to multiply the relevant virus in high amounts. For this reason, the cell line to be used is required to have high sensitivity to the relevant virus.^5,10,69,106^

### 4.5 Other Important Considerations

The aim of vaccine production is not only to protect public health and prevent diseases, but it also has a commercial concern, especially for private companies, and the companies that produce it also aim to make a profit.^18,75,107^ For this reason, in order to achieve high efficiency in the shortest time, the expression platform to be used for vaccine production is required to have features such as high accuracy expression capacity, ability to be successfully propagated in serum-free media, short doubling time, high maximum cell concentration, and ability to reach high viral titers.^4,15,17,18,69,108^ At the same time, since some cell lines are patented within the scope of intellectual property rights, their use in commercial production creates additional costs, and this should be taken into consideration when choosing a cell line.^97^ In addition, the origins of some cellular substrates, especially those of human origin, may pose ethical, psychological, and religious problems.^4,5^

## 5 Animal Cell Culture-Based Expression Platforms Used in Vaccine Production

Today, cell culture-based vaccines have been developed against many viral diseases. While some of these vaccines are only approved regionally and used within a certain geography, some have found global use by receiving approval from institutions that set world standards such as EMA and FDA. Many different cellular expressional platforms are used in the production process of all these vaccines (Table 2). In this section of this review, the expression platforms on which viral vaccines that are commercialized and used around the world are produced are mentioned.

**Table 2:** Approved Cell Culture-Based Viral Vaccines^a^

cell line	pathogen	vaccine type	vaccine trade name	manufacturer
Primary Monkey Kidney	Poliovirus	live-attenuated	Biopolio	Bharat Biotech
			Poliomyelitis Vaccine (Oral), Bivalent types 1 and 3	Serum Institute of India
			Bivalen Type 1 and 3 Oral Poliomyelitis Vaccines	PT BioFarma (Persero)
Primary Hamster Kidney	Japanese Encephalitis Virus	live-attenuated	CD.Jevax	Chengdu Institute of Biological Products
Primary Mouse Brain	Hantavirus	inactivated	Hantavax	Green Cross Pharma
CEF	Rabies	inactivated	Rabipur	Chiron Behring Vaccines Private/Novartis
			VaxiRAb N	Cadila Health Care
	Tick-borne encephalitis		TICOVAC	Pfizer
	Encepur			Bavarian Nordic
	Measles	live-attenuated	Priorix and Priorix-TETRA (only measles and mumps viruses are propagated on CEF in this vaccine)	Glaxo Smith Kline Biologicals
	Mumps		M-M-R II (only measles and mumps viruses are propagated on CEF in this vaccine)	MERCK
	Smallpox and Monkeypox		JYNNEOS	Bavarian Nordic
	Ebola/Ebola Virus	viral vector	Mvabea	Johnson & Johnson
WI-38	Rubella	live-attenuated	M-M-R II (only Rubella virus is propagated on WI-38 in this vaccine)	MERCK
	Adenovirus Type 4 and Type 7	live virus^b^	Adenovirus Type 4 and Type 7 Vaccine (only for military usage)	TEVA
MRC-5	Rabies	inactivated	IMOVAX Rabies	Sanofi Pasteur
	Hepatitis A		Havrix	Glaxo Smith Kline Biologicals
			AVAXIM	Sanofi Pasteur
	Measles	live-attenuated	MMR Tresivac (only measles and rubella viruses are propagated on MRC-5 in this vaccine)	Serum Institute of India
	Rubella		Priorix and Priorix-TETRA (only rubella and varicella viruses are propagated on MRC-5 in this vaccine)	Glaxo Smith Kline Biologicals
	Varicella zoster		SKYVaricella and SKYZoster	SK Bioscience
			VARIVAX	MERCK
			Varilrix	Glaxo Smith Kline Biologicals
	Poliovirus		Polio Sabin One and Three	Glaxo Smith Kline Biologicals
Vero	Poliovirus	inactivated	IPOL/IMOVAX Polio	Sanofi Pasteur
			Poliovac	Serum Institute of India
			Picovax	AJ Vaccines
			Poliomyelitis Vaccine (Vero Cell), inactivated, Sabin Strains	Sinovac Biotech
			Eupolio Inj.	LG Chem
	Japanese Encephalitis Virus		JEEV	Biological E. Limited
			IXIARO	Valneva
	Rabies		RABIVAX-S	Serum Institute of India
			VERORAB	Sanofi Pasteur
	SARS-CoV-2		CoronaVac	Sinovac
			Covaxin	Bharat Biotech
			KoviVac	Chumakov Center
			Turcovac	Health Institutes of Turkey and Dollvet
			FAKHRAVAC	Organization of Defensive Innovation and Research (Iran)
			QazCovid-in	Kazakh Research Institute for Biological Safety Problems
			KCONVAC	Shenzhen Kangtai Biological Products
			CovIran BAREKAT	Shifa Pharmed Industrial Group Company
			Covilo (BBIBP-CorV)	Sinopharm
			VLA2001	Valneva
	Poliovirus	live-attenuated	OPV/Opvero	Sanofi Pasteur
			Novel Oral (nOPV) Polio vaccine Monovalent type 2	PT BioFarma (Persero)
	Rotavirus		RotaRIX	Glaxo Smith Kline Biologicals
			RotaTeq	MERCK
			ROTASIIL	Serum Institute of India
			ROTAVAC	Bharat Biotech
	Dengue Fever/Dengue Virus	live-attenuated (Recombinant Chimeric Virus)	Dengvaxia	Sanofi Pasteur
	Japanese Encephalitis Virus		IMOJEV	GPO-Merieux Biologicals Products and Sanofi Pasteur
	Smallpox	live virus^c^	ACAM 2000	Sanofi Pasteur
	Ebola/Ebola Virus	viral vector	Ervebo	MERCK
MDCK	Influenza	inactivated	SKYCellflu	SK Bioscience
			Flucelvax	Seqirus
CHO	Hepatitis B	recombinant subunit	PreHevbrio	VBI Vaccines
	Varicella zoster		Shingrix	Glaxo Smith Kline Biologicals
	SARS-CoV-2		Zifivax (ZF2001)	Anhui Zhifei Longcom Biopharmaceutical
			Soberana 02/PastoCovac	Finlay Institute (Cuba) and Pasteur Institute of Iran
			V-01	Livzon Pharmaceutical Group
			MVC–COV1901	Medigen
			NVSI-06–08	National Vaccine and Serum Institute (China)
			Razi Cov Pars^d^ (only S-trimer is expressed in CHO)	Razi Vaccine and Serum Research Institute
Sf9	Influenza	recombinant subunit	Flublok	Sanofi Pasteur
	SARS-CoV-2		Nuvaxovid/Covovax/TAK-019	Novavax (Covovax manufactured with Serum Institute of India) (TAK-019 manufactured with Takeda)
			VidPrevtyn Beta	Sanofi Pasteur and Glaxo Smith Kline Biologicals
High 5 cells	Human Papilloma Virus (HPV)	VLP	CERVARIX	Glaxo Smith Kline Biologicals
	SARS-CoV-2	recombinant subunit	SpikoGen (Covax-19)	Vaxine and CinnaGen
HEK-293	SARS-CoV-2	viral vector	INCOVACC (BBV-154)	Bharat Biotech
			Convidecia	CanSino Biotech
			Sputnik V	Gamaleya
			Vaxzevria/Covishield	Oxford University/AstraZeneca (Covishield manufactured with Serum Institute of India)
		recombinant subunit	SKYCovione	SK Bioscience
			Razi Cov Pars^d^ (S1 and S2 are expressed in HEK)	Razi Vaccine and Serum Research Institute
PER.C6	Ebola Virus	viral vector	Zabdeno	Johnson & Johnson
	SARS-CoV-2		JCOVDEN	

a Sources: World health organization (WHO): VacciPROFILE database and vaccine product information sheets, European medicines agency (EMA): vaccine product information sheets, Centers for disease control and prevention (CDC): vaccine by disease database, Food and Drug Administration (FDA): vaccines highlights of prescribing information sheets.

b These adenoviruses (type 4 and type 7) are not attenuated.

c It is replication-competent live vaccina virus.

d Information about the vaccine expression platform is taken from.^109^

### 5.1 Primary Cultures and “CEF”

Today, primary cell cultures are still preferred in the production of viral vaccines. Although cultures such as primary mouse brain,^110^ primary monkey kidney,^5^ and primary hamster kidney^111^ are used for this purpose, the most preferred and accepted by authority organizations is the CEF culture.^5^ This is because CEF cultures can be obtained more easily in terms of cost and availability compared to other primary cultures used in vaccine production. CEF cultures have been involved in vaccine production for many years because they can be easily obtained from SPF eggs.^15,112^ M199, which is one of the general basal media developed for cell cultures as it has developed parallel to the evolution of cell culture technology, was actually developed for the use of CEF cells in serum-free vaccine production.^113^ CEF culture for vaccine production is created by taking the embryo from 10 to 12 day old SPF eggs with aseptic techniques, first dissociate it with mechanical and then enzymatic processes, and then placing the resulting cells in culture dishes and culture them.^5,11,112^ Compared to egg-based production, the production of more doses of vaccine is achieved by sacrificing a single embryo. It has been observed that the CEF culture, which must be re-established at the end of a certain production cycle, can be passaged 20–22 times before senescence.^112^ However, in the guidelines published by organisations that determine the regulations in vaccine production such as FDA, EMA, ICH, and World Health Organization (WHO), it is not recommended to passage CEF cultures to be used in vaccine production more than 3 times.^12,112,114^ The reason for this is the decrease in cellular proliferation as the cell character changes after the number of passages and the resulting decrease in virus production efficiency.^5,114^

### 5.2 WI-38

It was obtained from the lung tissue of a 12–13 week old female fetus in 1962 by Leonard Hayflick and his working group. This cell line with fibroblastic cell morphology is the first human diploid cell line used in vaccine production. This cell line was isolated at the Winstar Institute and named “WI-38” according to the sequence number in the serial experiments^115^ carried out to develop human diploid cell lines.^116^ This cell line has been shown to have a doubling time of approximately 24 h and can be maintained for up to 50 ± 10 passages.^117^ Afterward, these cells enter senescence according to the “Hayflick limit” and their characters vary greatly.^116^ For this reason, it is not recommended to use this cell line in viral vaccine production at advanced passage numbers.^12^ WI-38 cell line; it has been used in the development or direct production of vaccines against polio, measles, adenovirus, and rabies. In addition, this cell line has been frequently preferred, especially in attenuating viral strains used in production.^4,5,15^

### 5.3 MRC-5

Following the success of WI-38 in vaccine production, another human diploid cell line, MRC-5, was created by J. P. Jacobs and his team in 1966. Similar to WI-38, the MRC-5 cell line was obtained from the lung tissue of a 14-week-old male fetus. It was named MRC-5 because the place where this cell line was obtained was the Medical Research Council in England.^118,119^ This cell line also has a fibroblastic cell morphology, has a doubling time of approximately 34 h,^120^ and has been shown to be passaged approximately 45 times before going to senescence.^118^ In terms of vaccine production, a slowdown in the growth of cultures with passage numbers above 20 was observed.^118,121^ The MRC-5 diploid cell line is still the most used human cell line in vaccine production and viral infection studies, as its safety has been proven by all characterization studies and a sufficient number of standardized cell banks have been created.^12,121,122^ However, this cell line is sensitive to many different types of viruses^4,15,123,124^ and, like other continuous cell lines, can be made capable of interacting with viruses to which it is not sensitive through advanced cell engineering studies. In this sense, MRC-5 cells,^125,126^ which are not sensitive to the SARS-Cov-2 virus, were made sensitive to this virus by changing them to express the ACE2 receptor. Uemura et al. (2021), although it was originally aimed at antiviral drug trials, from the perspective of viral vaccine production, the potential for these modified MRC-5 cells to be used in the production of Covid-19 vaccines should be considered.^122^

### 5.4 Vero

Vero, a continuous cell line, was created by Yasumura and Kawakita at Chiba University in Japan in 1962 as a result of spontaneous transformation of cells in primary kidney culture obtained from an adult female African Green Monkey (*Chlorocebus aethiops*).^72^ Its name was created by the people who created this cell line by combining the words “Verda Reno”, which means green kidney in Esperanto.^127^ This cell line was the first continuous cell line approved and licensed for use in vaccine production and is still the most preferred cell line for the development and production of many vaccines today. One of the biggest reasons for this is that this cell line has a wide spectrum of viral sensitivity, including polio, hepatitis A, influenza, rabies, and yellow fever viral agents.^72,85,126^ Additionally, this cell line is a cell line that has been recommended by WHO for use in vaccine production for years because its effectiveness and safety have been proven. For this reason, the Vero cell line is used as the expression platform in commercial vaccines developed against many different viruses. In this respect, it is a cell line that can be considered a “*star player*” in cell culture-based viral vaccine production.^12,72,85,128,129^

Vero is a cell line that originally showed adherent growth, had a doubling time of approximately 24 h, and had an epithelial cell morphology.^130^ This cell line can also be adapted to reproduction in suspension using dynamic culture systems and serum-free culture media. However, as shown in studies, the doubling time of Vero cells adapted to suspended reproduction vary to be longer (more than 40 h).^84,131^ According to the culture conditions specified by WHO, it is not recommended to use cultures with advanced passage numbers in production, as the cell character changes greatly after the 150^th^ passage and acquires high tumorigenic properties. For this reason, cultures with early passage numbers are preferred in vaccine production in terms of safety and efficiency.^12,131,132^ However, Shen et al. (2019), it was determined that the population of Vero cells suspended in serum-free medium and adapted to reproduction was not tumorigenic when the population was evaluated at passage number 163.^131^ In another study conducted by Manohar et al. (2008), it was determined that the tumorigenicity of Vero cultures with passage number more than 200 increased depending on the passage number.^132^ In addition to all this, with a large number of cell engineering studies, especially using the CRISPR/Cas9 system, viral susceptibility was increased by knocking out various genes in Vero cells, and a significant increase in viral titers was observed in experiments with various viruses using edited cells.^133,134^

### 5.5 MDCK

The MDCK cell line was developed by Stewart Madin and Norman Darby in 1958. This cell line, which was created by continuing the primary culture of the kidney taken from an adult female Cocker Spaniel dog (*Canis lupus familiariz*), was also formed as a result of spontaneous immortalization.^135,136^ Its naming was made as “Madin-Darby Canine Kidney”, that is, MDCK for short, to include all the surnames of the people who developed it and the tissue from which it originated.^5,82,136,137^ The MDCK cell line originally has an epithelial morphology, shows adherent growth, and has a doubling time of 20–25 h.^138,139^ Additionally, it has been stated that the doubling time of this cell line, which can be adapted to suspension culture, varies between 20 and 40 h.^4,140−142^ Studies have reported that the reason for this variability is due to the operating parameters in different bioreactor systems.^140^ However, similar to Vero, the use of cells with high passage numbers is not preferred in production using MDCK cells.^12^ The reason why this cell line is preferred especially in viral vaccine production is that it is highly sensitive to almost all types of influenza viruses (both avian and human). In this respect, it is a frequently preferred expression platform in influenza virus studies, vaccine development processes and vaccine production.^5,137,143^ However, in order to improve the production process, a protease enzyme must be added to the production medium to convert hemagglutinin to its active form during the production process of influenza viruses.^144^ To eliminate this, there are studies conducted with MDCK cells modified to express TMPRSS2 (transmembrane protease, serine S1 family member 2)^145^ and HAT (human airway trypsin like protease) proteases, which altering hemagglutinin to the active form in natural influenza infection.^146^

### 5.6 CHO

This cell line was originally developed in 1956 by Thedore Puck and his working team from the ovarian tissue of an adult female Chinese Hamster (*Cricetulus griseus*). Similar to Vero and MDCK, this cell line formed by spontaneous immortalization is directly named CHO, which is short form of “Chinese Hamster Ovary”.^147,148^ Later, along with CHO-K1, which is considered ancestral because it was created by single cell cloning from the original CHO culture, many specific CHO cell subclones with different properties were developed over time with genetic expansion and adaptation methods to different culture conditions.^148,149^ The CHO cell line originally had an epithelial-like cell morphology and showed adherent growth. The adherent cells has a very short doubling time of 12–24 h.^150^ This cell line, like MDCK and Vero cells, can be adapted to suspension culture, and it has been shown that the doubling time of the adapted form can be similar or much lower than that of the adherent form.^76,151−153^

The CHO cell line is the most popular cell line in the bioprocessing industry. It is used in the production of many therapeutics, especially mAbs.^154^ The main reasons for this popularity are its suitability for the use of recombinant DNA techniques, its ability to perform the PTMs required for the therapeutic product to be actively functional in humans, and its high product yield. By using different production strategies, up to 10 g/L product can be obtained from the CHO cell line.^155,156^ In this respect, it has the potential to compete the production efficiency of some microbial systems.^154,155^ Additionally, this cell line is not sensitive to many human viruses thanks to its species barrier, and with cell engineering, CHO cells with increased resistance to possible contaminant viruses can be developed.^157^ While this becomes an advantage in the production of recombinant therapeutic proteins, it precludes the direct use of the CHO cell line in the production of viral vaccines, especially those based on the production of whole infective viruses.^105^ Although CHO cells do not have the required viral sensitivity, it has been shown that they can synthesize the structure of viral antigens with appropriate accuracy, especially due to PTM, which is similar to human cells, with recombinant DNA technology.^154−156^ Considering the other superior industrial properties it provides, it is especially preferred in the production of subunit viral vaccines.^158^ In addition to all these, in several clinical studies conducted to determine the effectiveness of hepatitis B vaccines, it has been shown that recombinant hepatitis B vaccines produced by using CHO cells are more effective in stimulating the immune system compared to traditional yeast expression systems.^159,160^

### 5.7 Sf9

This cell line originally originated from a cell line developed in 1970 by James E. Vaughn and his working group at the Insect Pathology Laboratory at the Beltsville Agricultural Research Center, Maryland, in the United States. The original cell line developed here was obtained from the ovarian tissue of the female fall armyworm (*Spodoptera frugiperda*) in pupal state.^161^ This original cell line resulting from spontaneous immortalization was named Sf21. In 1983, the Sf9 cell line was developed from this cell line by clonal selection.^161,162^ Since it was created by clonal selection, the morphology of Sf9 cells is more uniform than Sf21.^162,163^ Sf9 cell line is a cell line with an epithelial-like morphology and adherent growth. However, compared to other epithelial-like cell lines, the cells do not spread as much on the culture dish surface and have a more spherical appearance. The adherent form of this cell line has a doubling time of 24–32 h and can be easily adapted to suspension culture because it does not attach like general adherent morphology to the culture vessel surface.^163^ It has a similar doubling time as adapted for suspension growth.^164^

Because Sf9 cells are of insect origin, they are not susceptible to human viruses. Although this situation poses a problem for all types of vaccines based on virus production, it has the advantage of high production safety from the species barrier perspective.^95,162,164^ The Sf9 cell line is used in the production of recombinant DNA-based vaccines because they can synthesize immunologically suitable PTMs thanks to their expressional cell-machinery properties.^165^ For this purpose, baculovirus systems are used. In these systems, unlike the recombinant DNA technique, the gene that will synthesize the desired immunogenic part of the viral vaccine is delivered to the cell through baculoviruses instead of being directly transfected. In this way, Sf9 cells infected with baculoviruses carrying the relevant gene synthesize the desired antigen.^162,164,165^ It has been shown that production with Sf9, which has been approved for safety and has been in the biotechnology industry for many years, is contaminated with sf-rhabdovirus. Although several studies conducted on this situation, which creates safety concerns in terms of adventitious viral agents in production, have shown that this new type of virus does not replicate in cell lines of vertebrate origin, it should be addressed in more detail for eliminating concerns.^166,167^

### 5.8 Tn-5B1–4 (or High 5)

This continuous cell line originates from the original cell line developed by Robert Granados and his research team in 1986. This cell line was obtained from immature embryos in Cabbage Looper (*Trichoplusia ni*) eggs. Later, in 1994, the Tn-5B1–4 cell line was created by the same team at the Boyce Thompson Institute for Plant Research, New York, U.S.A., by clonal selection from this original cell line.^162,168,169^ It has been shown to have superior properties such as higher protein secretion and shorter doubling time (18–24 h) compared to other insect originated cell lines such as Sf9.^162,170^ In this respect, it is an advantageous expression platform, especially for recombinant protein production.^162^ This cell line was patented by Invitrogen and received the trade name High 5 for use in research and commercial production.^163^ This cell line shows adherent growth and, similar to Sf9, has a more spherical morphology as it does not spread much into the culture container, therefore it easily adapts to suspension culture.^162,163^ There are studies showing that as a result of adaptation, the doubling time decreases further (15–16 h).^171,172^ High 5 cell line, similar to Sf9, is used in the production of viral vaccines by recombinantly expressing the immunogenic viral parts desired to be included in the vaccine along with the use of baculovirus systems.^162,172,173^

### 5.9 HEK-293

HEK-293, which was first used for viral vaccine production with the Covid-19 pandemic, is a cell line currently used as an expression platform for CAR-T cell therapy, gene therapy, and the production of recombinant therapeutic proteins.^16^ Human origin allows this cell line to correctly perform PTMs in the human structure, eliminating possibilities such as undesirable immunological response and misfolding.^106,174^ Together with recombinant DNA technology, it is very suitable for the production of both therapeutic proteins and vaccine platforms such as subunit and VLP.^16,174−176^ Since it is of human origin, studies have shown that it is sensitive to viruses such as polio,^177^ influenza,^178^ and rabies,^179^ and it has been stated that it can be used as an expression platform for vaccines based on all virus production. It has also been shown that it can be infected with SARS-COV-2, albeit with low sensitivity.^180^ Additionally, this cell line is frequently preferred in modeling, toxicology, and drug testing studies.^106,174^

The HEK-293 cell line originates from a primary culture established by Alex Van der Eb in 1972 from the kidney cells of a female fetus. Later, this primary culture was used in studies conducted by Frank Graham to understand the cancer potential of adenoviruses in humans. In Graham’s studies originally carried out in 1973, the entire genome of Adenovirus Type 5 (AdV 5) was isolated and fragmented, and cells in primary kidney culture were transfected with these genome fragments. Since a continuous cell line was created in the 293^rd^ experiment in which these transfections were performed, its name was determined as HEK-293.^106,181^ With developing techniques in time, it has been shown that the HEK-293 cell line was immortalized by integrating an approximately 4.35 kbp long fragment containing the E1A and E1B gene regions into Chromosome 19 as a result of transfection with the AdV 5 genome.^182,183^ In this sense, the fact that HEK-293 cells have the E1A and E1B genes of AdV 5 and constantly express these genes makes them stand out specifically for the production of replication-deficient adenovirus and adeno-associated viral vector. In addition, these genes are interfering with the apoptosis process in the cellular cycle and ensuring immortality of HEK-293.^16,106,174,183^ Although the HEK-293 cell line is generally accepted to have an epithelial cell morphology showing adherent growth, studies have shown that this cell line also exhibits characteristics of neuron cells.^106,184^ It has been shown in many different studies that the doubling time of this cell line is between 30 and 40 h and that it can be easily adapted to suspension reproduction. It is also included in these studies that the version adapted to suspension has a similar or shorter doubling time.^183,185^ However, HEK-293 cells are known to be tumorigenic^100^ and in a study by Shen et al. (2008) conducted with nude mice, it was revealed that tumor formation was not observed when the passage number of HEK-293 cells was below 52, whereas tumor formation was observed when the passage number was above 65.^99^ Over the years, different HEK-293 cell derivatives with different cellular characters have been produced by different applications for different studies. The most well-known of these is HEK-293T, whose expression capacity has been increased by changing it to have the SV40 large T antigen.^174,175^

### 5.10 PER.C6

Another expressional platform that was commercially used for the first time in viral vaccine production with the Covid-19 pandemic is PER.C6. The original origin of this cell line is based on human embryonic retina (HER) cells isolated from the fetus in 1985.^186^ It received this name because it was developed by the same working group from the sixth cell clone of the cell line called PER.^187^ This cell line was created as a result of immortalization with the E1 gene region of adenovirus type 5, similar to HEK-293.^188,189^ However, the PER.C6 cell line was immortalized in a way that it does not contain the full-length E1 gene region, as in the HEK-293 cell line. The reason for this, minimize the formation of undesirable replication-competent viral vectors as a result of homologous recombination that may occur due to the overlapping sequences of the HEK-293 cellular genome, which contains the entire E1 gene region, with the adenoviral vector structure used during the production of the replication-defective viral vector.^108,190^ For this reason, PER.C6 was created with a DNA construct containing only nucleotides 459–3510 of the gene region encoding E1A and EB1 and containing human phosphoglycerate kinase as the promoter.^108,189,190^ The purpose of this is to develop the PER.C6 cell line directly for use in replication-deficient viral vector vaccines and gene therapy products for the biotechnology industry to ensure high production safety.^108,190^ In addition, since detailed records are kept from the development stage, full characterization is made, and the cell banking system is shown in detail, this cell line meets all the requirement determined by the regulatory institutions.^108,188^ In other studies, the oncogenicity and tumorigenicity of this cell line were evaluated using live cell injection, cellular DNA, cell lysate, and viral vector. While it has been shown that only high concentrations of live cell injection can induce tumor formation, it has been demonstrated that cell lysate, DNA and product did not trigger tumor formation at the injection site. These findings once again demonstrate the safety of this cell line.^73,191^ Designed specifically as an industrial production platform, the PER.C6 cell line is defined as “designer cell substrate” in the literature.^15^

It has been shown that PER.C6 cells have an adherent character, but can be easily adapted to suspension, and the doubling time is approximately 30–35 h.^189,192^ However, it has also been shown in these studies that this becomes shorter in subsequent passages.^193^ In the name of viral vaccine production, it has been shown that PER.C6 cells can be used in the production of adenovirus-based viral vector vaccines against viral agents such as SARS-Cov-19 and HIV,^186,194^ as well as being infected with viruses such as influenza^195,196^ and polio.^197^ This cell line, which is advantageous due to its human origin, can perform human PTMs.^198^ PER.C6 is a patented cell line with all rights belonging to Johnson & Johnson,^186^ and unlike HEK-293, another cell line used commercially in the production of replication-deficient adenoviral vector vaccine, licensing rights must be purchased. This situation creates a very high cost and restriction for other scientific studies. Accordingly, the HEK-293 cell line is more preferred in the production of adenoviral vectors, other viral vaccines and large-scale studies.^190^

### 5.11 Other Potential Cell Lines

All cellular expression platforms that have come into use in industrial viral vaccine production so far are summarized. Apart from these, there are different cell lines used in the production of different vaccines that have been developed and are still in the process of being approved as vaccine candidates. Many of these cell lines are called “designer” because they are designed directly for industrial production, such as PER.C6.^4,6,14,15^ Among these, the ones that have a high potential to be used in commercial production in the coming years are CAP,^199^ EB66,^4,15,200^ AGE1.CR,^4,6,15,201^ PBG.PK2.1,^202^ and DF-1.^203^ The idea has emerged that some other cell lines, which have been used in scientific studies for many years but have safety concerns,^10,23^ especially due to their cancer origin, may also be included in commercial production with the Covid-19 pandemic. This is because expression platforms such as HEK-293 and PER.C6, which have similar features and were previously the focus of such concerns, have been approved for use.^4,15,16^ In this respect, it is thought that human-origin Calu-3 cells, which are widely used for cell-virus interaction especially in Covid-19, may have the potential to be used in viral vaccine production when all safety conditions are met, although they are highly neoplastic, with developing techniques.^104,204^ A-549, which is a similar cell line and has been used in scientific studies for many years, is also known to be sensitive to many different viruses.^126^ However, it has been shown that it can also be used in the production of adenovirus,^9,24^ which has become popular again in recent years.^190^ A-549, which is also human origin, is a cell line with a very high potential for use after its reliability in viral vaccine production has been demonstrated in more detail.^87,103^ However, since only approved and commercially used cellular platforms are covered in this review, all these other potential vaccine expression platforms are not elaborated.

## 6 Future Perspective and Conclusions

The Covid-19 pandemic and other epidemics that occurred before have once again shown that it is essential for vaccines to reach all societies quickly. In addition to conventional vaccines, which have traditionally been used safely for many years, new high-tech vaccine platforms that can be produced much more quickly have been developed and these have been approved and put into use for the first time. Although some of these can be produced directly using cell-free systems, cellular expression platforms are needed, especially in the production of traditional ones and other new vaccine types. Among these platforms, continuous cell lines stand out especially due to the advantages they provide.

Considering the increasing world population and the potential for epidemic/pandemic diseases to occur, it is thought that the need for vaccines and biotechnological medicines will gradually increase. For this reason, the aim is to produce the maximum product in the shortest time in industrial production. However, considering the production quantities and costs of biopharmaceuticals, it becomes difficult for the entire population to access them. However, due to scarcity of world resources, it is becoming increasingly necessary for all biological production processes to be demonstrated not only for their safety and effectiveness, but also for their economic and ecological suitability. For this reason, while achieving maximum production efficiency with minimum resource input has become a goal, the time parameter is also quite critical. In addition, while planning all of these, the sources of all raw materials used, the production process, the processing steps and the environmental impact in the process until the final product is delivered to the consumer must also be taken into consideration in today’s world. Only in this way can the biopharmaceutical industry continue in a sustainable manner.

Cell culture systems, where the majority of viral vaccine production is carried out, are considered expensive and time-consuming systems with high input. Nucleic acid and synthetic peptide vaccines, which came into commercial use with Covid-19, provide advantages in terms of cost and time by mostly eliminating the need for expression systems. Synthetic peptide vaccines, in which no cellular system is used during the production phase, stand out more in this sense. However, they have disadvantages such as high use of chemicals in their production processes, energy input and requiring more special storage conditions in their transportation. In addition, there are problems in terms of effectiveness and safety for both vaccine types compared to conventional vaccine types. However, cell culture systems have been known to be effective and used safely for many years. In addition, there is a trend toward the use of continuous cell lines in the biopharmaceutical industry, as their safety has been proven thanks to the advanced techniques and detailed upstream and downstream processes in many years. Also, it is very advantageous that continuous cell lines can be grown in chemically defined culture media, free from expensive, ecologically, and ethically unsuitable components such as serum. With Covid-19, continuous cell lines, which are normally considered unsafe for use in production, have first begun to be used for commercially large-scale production. It is stated that both of these cell lines, which are produced for the first time especially for viral vaccine production, are of human origin and are preferred due to the advantages they provide in the biotechnology industry compared to other continuous nonhuman cell lines. In this sense, it is thought that in the coming years, the choices made in the selection of expression platforms in viral vaccine production will largely be in favor of these, both in terms of immunological accuracy and because they can reach commercially sufficient viral titers. Alternatively, it stands out because the production conditions in bioprocesses carried out with cell culture and the need for extreme conditions in the logistics of the products are lower. In this way, safe and effective vaccines will be produced in large quantities at lower cost by using cell culture technology, and industrial sustainability targets can be achieved. In addition to all these, with the development of new vaccine platforms similar to today in viral vaccine production, it is possible to transition to completely cell-free systems in the future with the discovery of more advanced technologies. While the all viral vaccines can be produced with cell-free systems in far future cell culture is remain for being model systems in the basic techniques used in scientific studies. Along with that it is thought that the use of cell lines along with cell culture technology will still be necessary in the long term in gene therapy, cancer treatment, and tissue engineering applications, which are within the scope of personalized medicine applications developed within the biopharmaceutical industry.
